# Myc/Max dependent intronic long antisense noncoding RNA, EVA1A-AS, suppresses the expression of Myc/Max dependent anti-proliferating gene EVA1A in a U2 dependent manner

**DOI:** 10.1038/s41598-019-53944-2

**Published:** 2019-11-21

**Authors:** Svenja E. Niehus, Aldrige B. Allister, Andrea Hoffmann, Lutz Wiehlmann, Teruko Tamura, Doan Duy Hai Tran

**Affiliations:** 1 Institut fuer Biochemie, OE4310, Medizinische Hochschule Hannover, Carl-Neuberg-Str. 1, D-30623 Hannover, Germany; 2 Klinik für Orthopädie OE8893, Medizinische Hochschule Hannover, Stadtfelddamm 34, D-30625 Hannover, Germany; 3 Zentrale Forschungseinrichtung Genomics OE 9415, Medizinische Hochschule Hannover, Carl-Neuberg-Str. 1, D-30623 Hannover, Germany

**Keywords:** Cancer, Molecular biology

## Abstract

The Myc gene has been implicated in the pathogenesis of most types of human cancerous tumors. Myc/Max activates large numbers of pro-tumor genes; however it also induces anti-proliferation genes. When anti-proliferation genes are activated by Myc, cancer cells can only survive if they are downregulated. Hepatocellular carcinoma (HCC) specific intronic long noncoding antisense (lnc-AS) RNA, the EVA1A-AS gene, is located within the second intron (I2) of the EVA1A gene (EVA-1 homolog A) that encodes an anti-proliferation factor. Indeed, EVA1A, but not EVA1A-AS, is expressed in normal liver. Depletion of EVA1A-AS suppressed cell proliferation of HepG2 cells by upregulation of EVA1A. Overexpression of EVA1A caused cell death at the G2/M phase via microtubule catastrophe. Furthermore, suppressed EVA1A expression levels are negatively correlated with differentiation grade in 365 primary HCCs, while EVA1A-AS expression levels are positively correlated with patient survival. Notably, both EVA1A and EVA1A-AS were activated by the Myc/Max complex. Eva1A-AS is transcribed in the opposite direction near the 3′splice site of EVA1A I2. The second intron did not splice out in a U2 dependent manner and EVA1A mRNA is not exported. Thus, the Myc/Max dependent anti-proliferating gene, EVA1A, is controlled by Myc/Max dependent anti-sense noncoding RNA for HCC survival.

## Introduction

Noncoding RNAs regulate a broad spectrum of cellular processes, including oncogenic signaling. Recent deep sequencing studies determined that the number of long noncoding RNAs is 2- to 3-fold greater than protein coding RNAs^1^. Furthermore, by analyzing RNA-seq data from 20 HCC patients, 8603 long noncoding RNA (lncRNAs) were recently identified^2^. Notably, approximately 76% of these were unannotated^2^, suggesting that the expression of lncRNAs is highly cell type and cancer type specific.

Comprehensive expression analysis of the Encyclopedia of DNA Elements Consortium (ENCODE) indicated that three-quarters of the human genome is capable of being transcribed, but only about 1.5% of this fraction contains the genome code for proteins^3,4^.

We have recently shown that upon depletion of THOC5, a member of the TREX (transcription/export) complex 396 genes were commonly downregulated in HCC cell lines, Huh7 and HepG2, and those affected cells then underwent apoptosis^5^. Here, depletion of Linc00176, one of the THOC5 target long noncoding RNAs, resulted in cell death in HCC^6^. We then proceeded to search for THOC5 target long noncoding RNAs for HCC maintenance.

In this study we have characterized one of these THOC5 target antisense (AS) long noncoding genes, EVA1A (eva-1homolog A)/TMEM166 (transmembrane protein 166)-antisense (EVA1A-AS). Both EVA1A and EVA1A-AS genes are activated by the Myc/Max complex that is the most commonly overexpressed gene in human cancers. We show that the intronic AS lncRNA, EVA1A-AS, suppressed EVA1A expression and EVA1A is highly expressed in normal liver, but not in HCCs. Overexpression of EVA1A as well as depletion of EVA1A-AS induced inhibiting cell proliferation and cell death in HCC. Here EVA1A-AS suppressed splicing of EVA1A intron 2 in a U2 dependent manner. These data imply that EVA1A-AS participates in the survival of HCC that expresses EVA1A.

## Results

### EVA1A-AS is expressed in HepG2 cells, but not in normal hepatocytes and suppresses EVA1A expression

**Figure 1:**
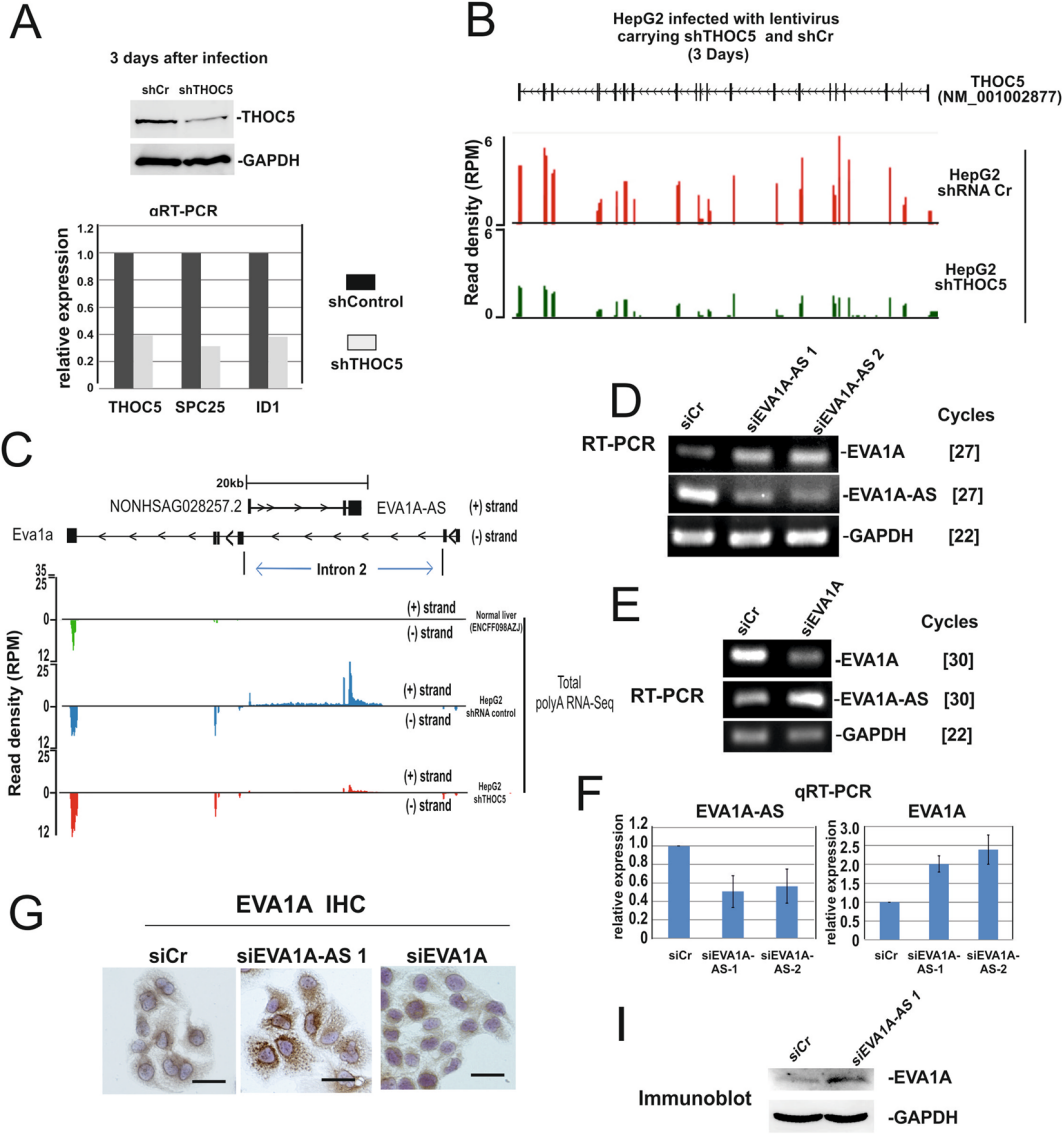
EVA1A-AS is expressed in HepG2 cells, but not in normal hepatocytes and suppresses EVA1A expression. HepG2 cells were infected with lentivirus carrying shTHOC5 (shTHOC5) or sh control (shCr) for three days. (**A**) Cell lysates were applied for THOC5 specific immunoblot. GAPDH specific immunoblot was performed as loading control. RNAs were isolated from a sister culture and applied for THOC5, SPC25, ID1 specific qRT-PCR. (**B**) RNA sequence data of THOC5. SeqMonk was used to quantitate and visualize the data. Read density is shown as reads per million (RPM). (**C**) Predicted EVA1A-AS (NONHSAG028257.2) (+strand) and EVA1A (−strand) in HepG2 cells infected with lentivirus carrying shRNA control or shTHOC5. Total RNA-seq datasets from human liver tissue (ENCFF098AZJ) were generated by the ENCODE Consortium and were aligned to the reference human genome (GRCh38). Black box: exon. (**D**) RNAs were isolated from HepG2 treated with siCr, siEVA1A-AS-1, or siEVA1A-AS-2 and were supplied for EVA1A, EVA1A-AS or GAPDH specific semi-quantitative RT-PCR. []: number of PCR cycles. (**E**) RNAs were isolated from HepG2 cells treated with siCr, or siEVA1A and supplied for EVA1A-AS, EVA1A or GAPDH specific semi-quantitative RT-PCR. []: number of PCR cycles. (**F**) RNAs were isolated from sister culture of (**D**) and applied for EVA1A-AS, EVA1A specific q-RT-PCR. Three independent experiments were performed. Bars represent +/−SD. (**G**) Cell extracts from a sister culture of (**D**) were applied for EVA1A and GAPDH specific immunoblot.

We have previously shown that depletion of THOC5, an mRNA export complex protein, in HCC downregulates only a subset of genes, but causes cell death^5^. One of the essential THOC5 target genes for HCC maintenance is a long noncoding RNA, Linc00176^6^. These data imply that additional THOC5 target long noncoding RNAs may participate in HCC development and survival. To identify further THOC5 target long noncoding RNAs, HepG2 cells were infected with lentivirus carrying shTHOC5 or shCr (control) for 3 days. Under these conditions, THOC5 protein was downregulated to approximately 50% (Fig. 1A) and previously identified THOC5 target coding genes, inhibitor of DNA binding 1 (ID1) and NDC80 kinetochore complex component (SPC25), were also downregulated more than 2-fold (Fig. 1A). RNAs were then applied for RNA sequencing. The sequencing data of THOC5 revealed that all exon reads were clearly suppressed in THOC5 depleted cells (Fig. 1B).

The 3208 annotated long noncoding RNAs were downregulated more than 2-fold upon depletion of THOC5. These long noncoding RNA transcripts were further filtered for the following criteria: 1) expression of more than 100 FPKM (Fragments Per Kilobase of Exon Per Million Fragments Mapped) in shCr virus infected HepG2, 2) predicted length of RNA more than 500 nucleotides (nt), 3) expression of less than 50 FPKM in other tissues (Human BodyMap 2.0 data from Illumina). The 6 lncRNAs, namely NONHSAG028257.2, NONHSAG094271.1, NONHSAG030119.2, NONHSAG075906.1, NONHSAG015030.2, and NONHSAG035576.2, were selected using these criteria. We then further characterized the 2 long antisense ncRNAs (NONHSAG028257.2, and NONHSAG094271.1). NONHSAG028257.2 is an antisense noncoding RNA to the EVA1A gene, whereas NONHSAG094271.1 is an antisense noncoding RNA to the KRT19P1 pseudogene with unknown function. Since EVA1A is shown as anti-proliferating factor gene^7^, we examined the role of “NONHSAG028257.2” EVA1A antisense (AS) noncoding RNA in HCC (Fig. 1C). EVA1A-AS is located in the second intron (I2) of the EVA-1A gene (Fig. 1C). Notably, our sequencing data reveals that depletion of THOC5 reduced the expression level of EVA1A-AS, whereas in contrast EVA1A expression is enhanced (Fig. 1C), suggesting that these two genes may regulate each other. Indeed, depletion of EVA1A or EVA1A-AS by treatment with specific siRNAs in HepG2 cells enhanced the expression level of EVA1A-AS or EVA1A, respectively (Fig. 1D–F). EVA1A protein is also upregulated in EVA1A-AS depleted cells and the protein was expressed at low level in siCr treated HepG2 cells (Fig. 1G), indicating that EVA1A-AS is a suppressor for EVA1A expression.

### Depletion of EVA1A-AS in HepG2 cells induced lipid droplet accumulation and stopped the cell cycle, but additional depletion of EVA1A rescued cell proliferation

**Figure 2:**
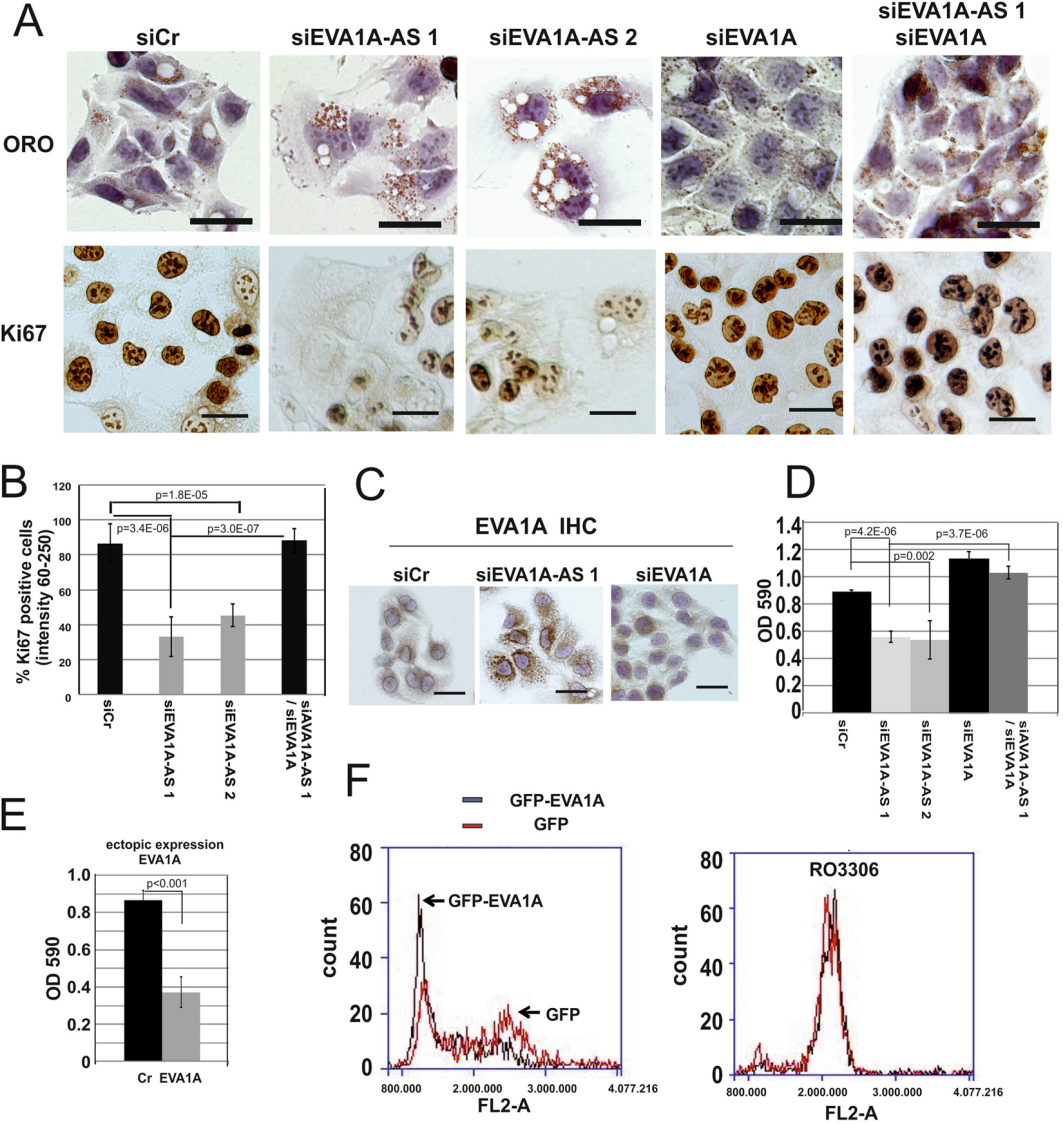
Depletion of EVA1A-AS in HepG2 cells induced lipid droplet accumulation and stopped cell cycle. (**A**) HepG2 cells were chemically fixed 3 days after transfection with siCr, siEVA1A-AS 1, siEVA1A-AS 2 and/or siEVA1A and then applied for oil red O (ORO) or Ki67 specific immunohistochemical staining. Bars represent 40 µm. Three independent experiments were performed and representative images are shown. (**B**) To quantitate the intensity of Ki67 staining, reciprocal pixel intensity was determined by subtracting the Ki67 intensity from the maximum pixel intensity in white unstained areas (as measured by the mean intensity function in the Nikon NIS elements D 3.0 Software). The mean percentage of dark staining (positive: reciprocal intensity 60–250) nuclei is shown. Bars represent +/−SD. At least 200 cells from 3 images were quantitated per experiment. p value: t-test. (**C**) Endogenous EVA1A was immunohistochemically stained in HepG2 cells after transfection with siCr, siEVA1A-AS, or siEVA1A. Bars represent 40 µm. (**D**) Sister culture of (A) was stained with crystal violet and measured density by OD590. (**E**) EVA1A was ectopically expressed in HepG2 cells and three days after transfection cells were fixed and stained with crystal violet. (**F**) HepG2 cells were transfected with GFP or GFP-EVA1A in the presence or absence of CDK1 inhibitor, RO3306. Fixed cells were stained with propidium iodide (PI) and analysed by Accuri-C6 flow cytometer (BD Biosciences) under standard settings for detecting PI (FL-2) using a 488 nm laser.

Upon depletion of EVA1A-AS, large vacuoles and lipid droplets were observed within 3 days (Fig. 2A). Lipid droplets were not detected within the vacuoles. Importantly, these cells become significantly larger than cells transfected with siCr, and these large cells simultaneously lose the expression of proliferation marker Ki67 (Fig. 2A,B). Upon additional depletion of EVA1A, siEVA1A-AS transfected cells significantly regained Ki67 expression (Fig. 2A,B, p = 3.0E-07). Here, upon depletion of EVA1A alone, the phenotype of HepG2 was not significantly altered (Fig. 2A). To examine the subcellular localization of endogenous EVA1A, we stained HepG2 cells with anti EVA1A antibody before and after depletion of EVA1A-AS. Endogenous EVA1A is located mainly at the perinuclear region in untreated cells, but upon depletion of EVA1A-AS, EVA1A is distributed throughout the cytoplasm in punctate forms that are similar to lipid droplets by ORO staining (Fig. 2A,C), and EVA1A is partially co-localized with lipid droplets.

To examine whether EVA1A and EVA1A-AS control cell growth, we examined cell growth based on crystal violet staining. In agreement with Ki67 staining data, upon depletion with EVA1A-AS, not EVA1A, cell growth was approximately 50% reduced within 3 days (Fig. 2D). Cell growth of siEVA1A-AS transfected cells was recovered when cells were further transfected with siEVA1A (Fig. 2D), while overexpression of EVA1A suppressed cell growth (Fig. 2E).

To examine which cell cycle phase is influenced by overexpression of EVA1A, propidium iodide (PI) labeled cells were analyzed by FACS (Fig. 2F). G2 peak of EVA1A overexpressing HepG2 cells was drastically reduced (Fig. 2F), suggesting that EVA1A caused G1-S arrest or cell death in G2-M phase. We then examined cell cycle of EVA1A overexpressing cells and control HepG2 cells in the presence or absence of the blocker for G2/M phase CDK-1 kinase inhibitor, RO3306 (5 µM). Both cells went G2 phase (Fig. 2F), suggesting that EVA1A overexpressing cells did not go to G1-S arrest.

### Ectopic expression of EVA1A in HCC cells caused cell death after G2 phase

**Figure 3:**
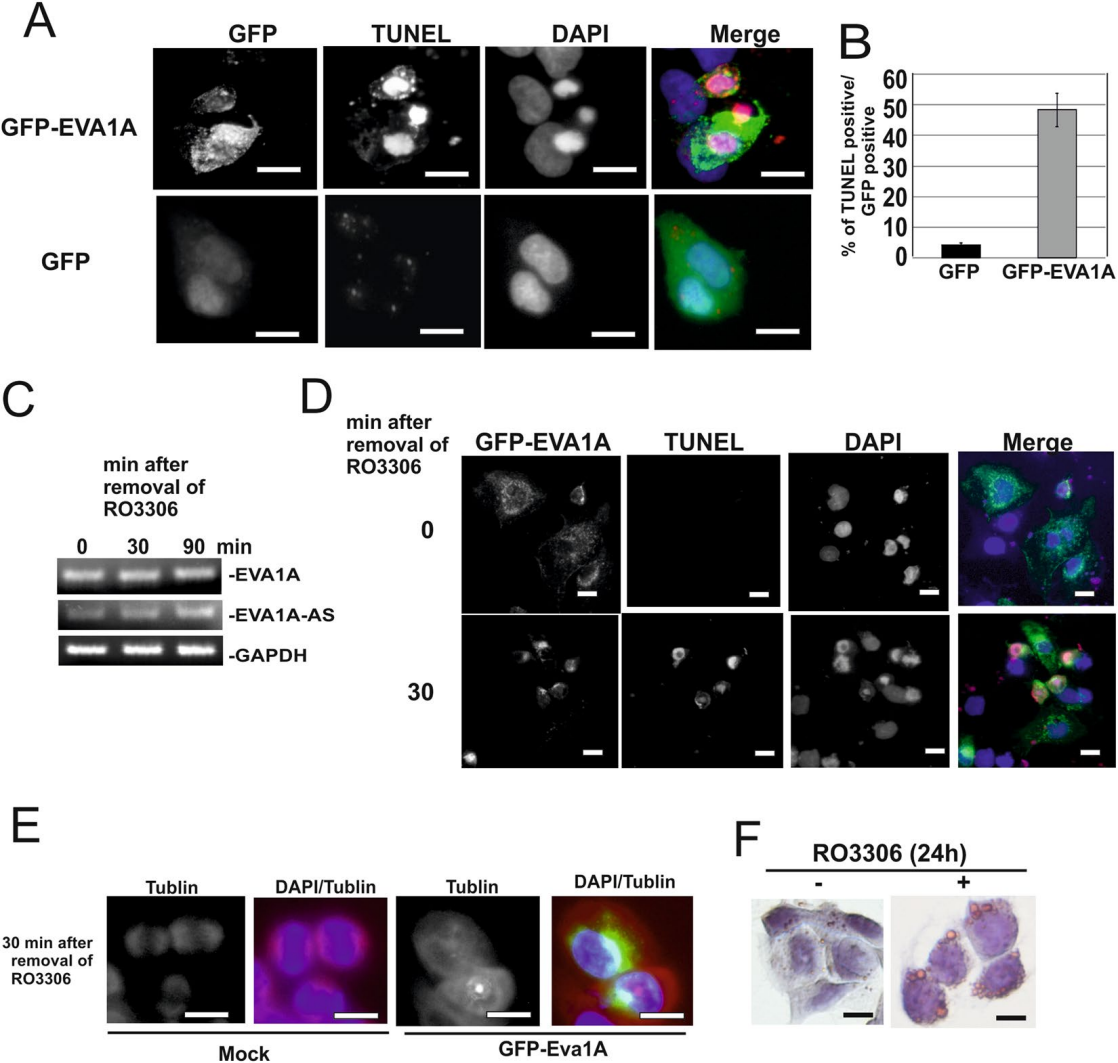
Ectopic expression of EVA1A in HCC cells caused cell death. (AB) GFP-EVA1A or GFP was overexpressed in HepG2 cells and applied for TUNEL (red), and DAPI (blue) staining. Bars represent 40 µm. (**A**) The ratio between GFP: TUNEL positive cells was determined. (**B**) (**C**) HepG2 cells were treated with CDK1 kinase inhibitor RO3306 (5 µM) for 24 h. Total RNAs obtained from cells after removal of inhibitor (0, 30, and 120 min) were applied for EVA1A, EVA1A-AS or GAPDH specific semi quantitative RT-PCR as indicated. (**D**,**E**) HepG2 cells were transfected with GFP-EVA1A cDNA and then treated with CDK1 kinase inhibitor RO3306 (5 µM) for 24 h. Cells were fixed 0 or 30 min after removal of inhibitor. Cells were stained by DAPI and TUNEL (**D**) or Tubulin (**E**) antibody and DAPI. Three independent experiments were performed and representative images are shown. Bars represent 40 µm. (**F**) HepG2 cells were incubated with (+) or without (−) RO3306 for 24 h. Cells were stained by ORO. Bars represent 40 µm.

To examine whether overexpression of EVA1A causes cell death in HepG2 cells, we next expressed GFP-EVA1A in HepG2 cells. Half of the GFP-EVA1A positive cells became TUNEL positive within 24 h after transfection, while expression of GFP alone resulted in less than 5% TUNEL positive cells (Fig. 3A,B). We obtained similar data using Myc-tagged EVA1A (SI Fig. 1A). Apoptosis markers, such as cleavage of PARP or caspase 3 were not detected in EVA1A overexpressing HepG2 cells, and MLKL was not phosphorylated in these cells (SI Fig. 2).

To examine whether EVA1A exerted an influence on the transition of G2/M phase, EVA1A overexpressing cells were treated with CDK-1 kinase inhibitor, RO3306 (5 µM) for 24 h, whereafter the inhibitor was removed. This treatment did not result in a change to the endogenous expression level of EVA1A, whereas RO3306 treatment suppressed EVA1A-AS expression. After removal of the inhibitor, however, the expression level of EVA1A-AS recovered within 90 min (Fig. 3C). Although RO3306 treated EVA1A positive cells increased in size, no TUNEL positive cells were observed (Fig. 3D), whereas removal of RO3306 induced TUNEL positive cells within 10 to 30 min (Fig. 3D). In addition, typical spindles were not observed by tubulin specific staining after the removal of the inhibitor in EVA1A overexpressing HepG2 cells (Fig. 3E), indicating that cells died during G2/M transition by microtubule catastrophe^8^. Notably, treatment of HepG2 cells with CDK1 inhibitor caused lipid droplet accumulation (Fig. 3F).

### EVA1A expression level is correlated with the differentiation grade of HCC

**Figure 4:**
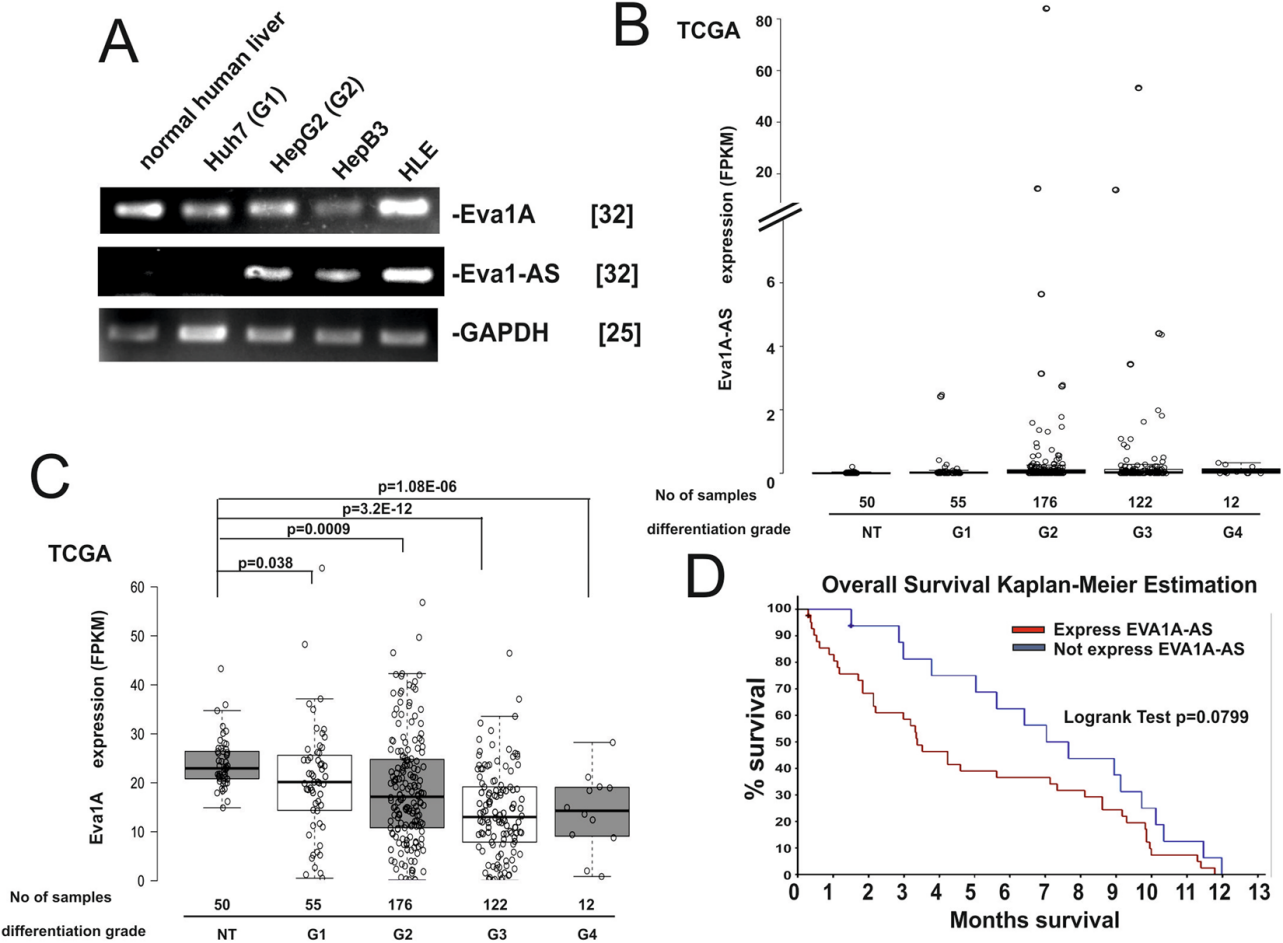
The EVA1A expression level is correlated with the differentiation grade of HCC. (**A**) Total RNAs from Huh7 (G1), HepG2 (G2), HepB3 (G3) or HLE cells were supplied for EVA1A, EVA1A-AS and GAPDH specific semi-quantitative RT-PCR. Normal human liver RNA was purchased from human normal hepatocyte (Origene, Maryland, USA). []: number of PCR cycles. Three independent experiments were performed. (**B**–**D**) Expression of EVA1A-AS (**B**) and EVA1A (**C**) in 365 primary HCCs (differentiation grade 1–4) and 50 normal liver samples. Data obtained from the cancer genome atlas (TCGA) (https://cancergenome.nih.gov/) shown as box blot. p value: t-test between G1, G2, G3 and G4 HCCs and 50 corresponding normal livers. (**D**) Correlation between EVA1A-AS expression level (FPKM >0 (n = 41): FPKM = 0 (n = 16)) and survival time (within 12 months) is shown using Kaplan-Meier estimation (Log rank test: p = 0.0799).

We next examined whether EVA1A and EVA1A-AS are expressed in normal liver and four HCC cell lines, HepG2, Huh7, Hep3B and HLE. EVA1A was expressed in all samples, however the expression level of EVA1A in HepG2 and Huh7 is approximately 5-fold less than in normal hepatocytes, while EVA1A-AS is expressed in HepG2, HepB3 and HLE cells (differentiation grade 2–3 (G2-G3) but not in Huh7 cells (differentiation grade 1 (G1)) or normal hepatocytes (Fig. 4A). In primary HCC EVA1A-AS expression was observed in only a subset of G2 and G3 HCCs (Fig. 4B), while EVA1A gene expression was significantly suppressed in G2 to G4 primary HCCs (p = 0.0009 - p = 3.2E-12) (Fig. 4C). These data suggest that EVA1A may act as a negative regulator of HCC survival. Indeed, the EVA1A-AS expression level is also significantly correlated with HCC patient survival time. We analyzed the data by means of the Kaplan-Meier estimation. Correlation between EVA1A-AS expression levels (FPKM >0 (n = 41): FPKM = 0 (n = 16)) and survival time (within 1 year) is significant (Fig. 4D, log rank test: p = 0.0799). These data suggest that EVA1A-AS participates in growth and/or survival of HCC in cell lines as well as in primary HCCs.

We next examined whether a point mutation of p53 correlates with EVA1A-AS expression. In HCC cell lines, HepG2 has p53 without a mutation and in Huh7 and HLE cells p53 is point mutated. EVA1A-AS is expressed in HepG2, and HLE, but not in Huh7 (Fig. 4A), indicating that EVA1A-AS expression is not correlated with a p53 mutation. In agreement with these data, in primary HCC 40–50% of p53 are mutated in both HCC with or without EVA1A-AS expression (Data obtained from the cancer genome atlas).

### Both EVA1A-AS and EVA1A are regulated by the Myc/Max complex

**Figure 5:**
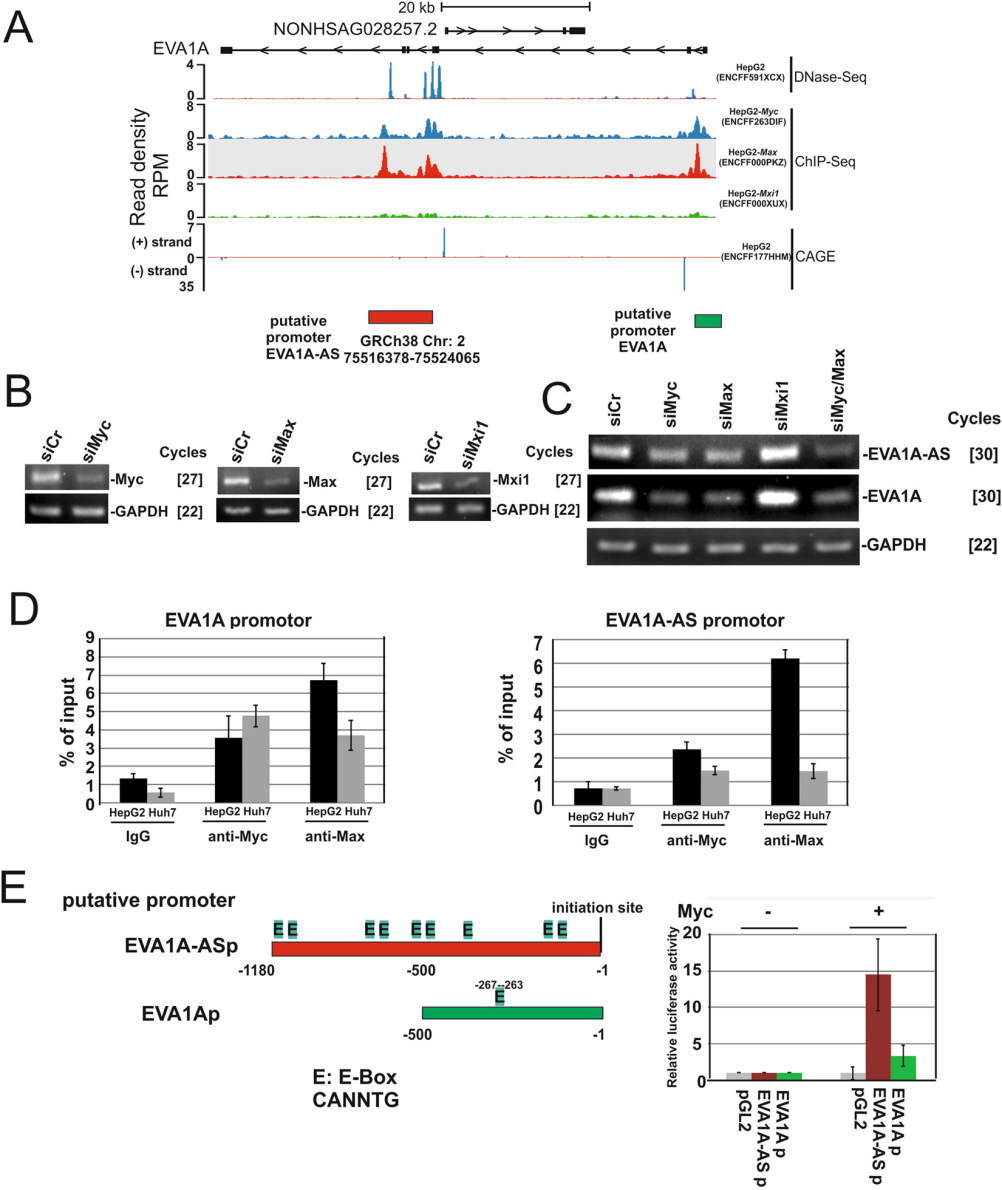
Both EVA1A and EVA1A-AS were regulated by Myc/Max. (**A**) DNase-sequencing (DNase-seq) datasets from HepG2 (ENCFF591XCX), ChIP-seq datasets HepG2 Myc (ENCFF263DIF), HepG2 Max (ENCFF000PKZ), and HepG2 Mxi1 (ENCFF000XUX), and cap analysis of gene expression (CAGE) data in HepG2 cells (ENCFF177HHM) generated by the ENCODE Consortium were aligned to the reference human genome (GRCh38). SeqMonk was used to quantitate and visualize the data. Yellow box: putative EVA1A-AS promoter; green box: putative EVA1A promoter. (**B**,**C**) siCr, siMyc, siMax, or siMxi1, were transfected in HepG2 cells and RNA was applied for Myc, Max, Mxi1, EVA1A-AS, EVA1A or GAPDH specific semi quantitative qRT-PCR as indicated. []: number of PCR cycles. Three independent experiments were performed and representative images are shown. (**D**) After cross-linking by adding formaldehyde, protein and DNA were extracted from HepG2 or Huh7 cells and the chromatin was sheared by sonication. Cell extracts and immunoprecipitates using Myc or Max antibodies or control IgG were analyzed by EVA1A (promoter region, −124 to −299) and EVA1A-AS (promoter region,−42 to −235) specific PCR (Table 1; ChIP). Numbers represent nucleotide numbers from the initiation site for each gene. Data represent percent (%) input of each PCR reaction. Three independent experiments were performed. (**E**) location of E-Box (E: CANNTG) in the putative promoter regions of EVA1A-AS (EVA1A-ASp) and EVA1A (EVA1Ap). Putative promoter region of EVA1A-AS and EVA1A fused luciferase and luciferase activity was examined in the presence or absence of Myc.

We next examined the manner in which EVA1A–AS is transcriptionally regulated. DNase-sequencing (ENCFF591XCX) and cap analysis of gene expression (CAGE) (ENCFF177HHM) data suggest that the promoter of EVA1A-AS is located on “GRCh38 Chr2: 75516378–75524065” in HepG2 (Fig. 5A, yellow box). ENCODE ChIP-seq data revealed that Myc (ENCFF263DIF) and Max (ENCFF000PKZ) bind to this position and Mxi1 (ENCFF000XUX), a negative regulator for Myc/Max^9^, binds to a much lesser extent (Fig. 5A). In agreement with these data, upon depletion of Max and/or Myc, the EVA1A-AS expression level is downregulated more than 4-fold (Fig. 5B,C), however depletion of Mxi1, suppressor of Max/Myc, upregulated EVA1A-AS expression (Fig. 5C). Strikingly, Myc- and Max-CHIP-seq data suggest that the EVA1A promoter is also regulated by Myc (Fig. 5A, green box). Upon knockdown of Myc and/or Max, EVA1A expression is downregulated. Depletion of Mxi1 results in upregulation of EVA1A (Fig. 5C).

To confirm these data, we next performed a Myc- and Max- specific chromatin immunoprecipitation (CHIP) assay. In HepG2 cells, Myc and Max bound to both proximal promoter regions of Eva1A and Eva1A-AS, while in Huh7 cells Myc and Max bound to EVA1A, but not to the EVA1A-AS promoter region (Fig. 5D). EVA1A-AS and EVA1A proximal promoter regions contain 9x and 1x E-BOX (CANNTG), respectively (Fig. 5E). To determine whether these putative promoters are indeed activated by Myc, both proximal promoter regions were fused to the luciferase gene and the reporter assay was performed in the presence or absence of Myc^10^. In the presence of Myc, EVA1A-AS and EVA1A putative promoters were activated 14- and 3.3 -fold in the absence of Myc, respectively (Fig. 5E).

These data imply that Myc/Max induces both the EVA1A and EVA1A-AS genes and since EVA1A is an anti-survival protein in HCC, EVA1A-AS suppressed the expression of EVA1A, resulting in the survival of HCC.

### EVA1A-AS suppressed EVA1A expression by inhibiting the splicing of intron 2 (I2) of EVA1A

**Figure 6:**
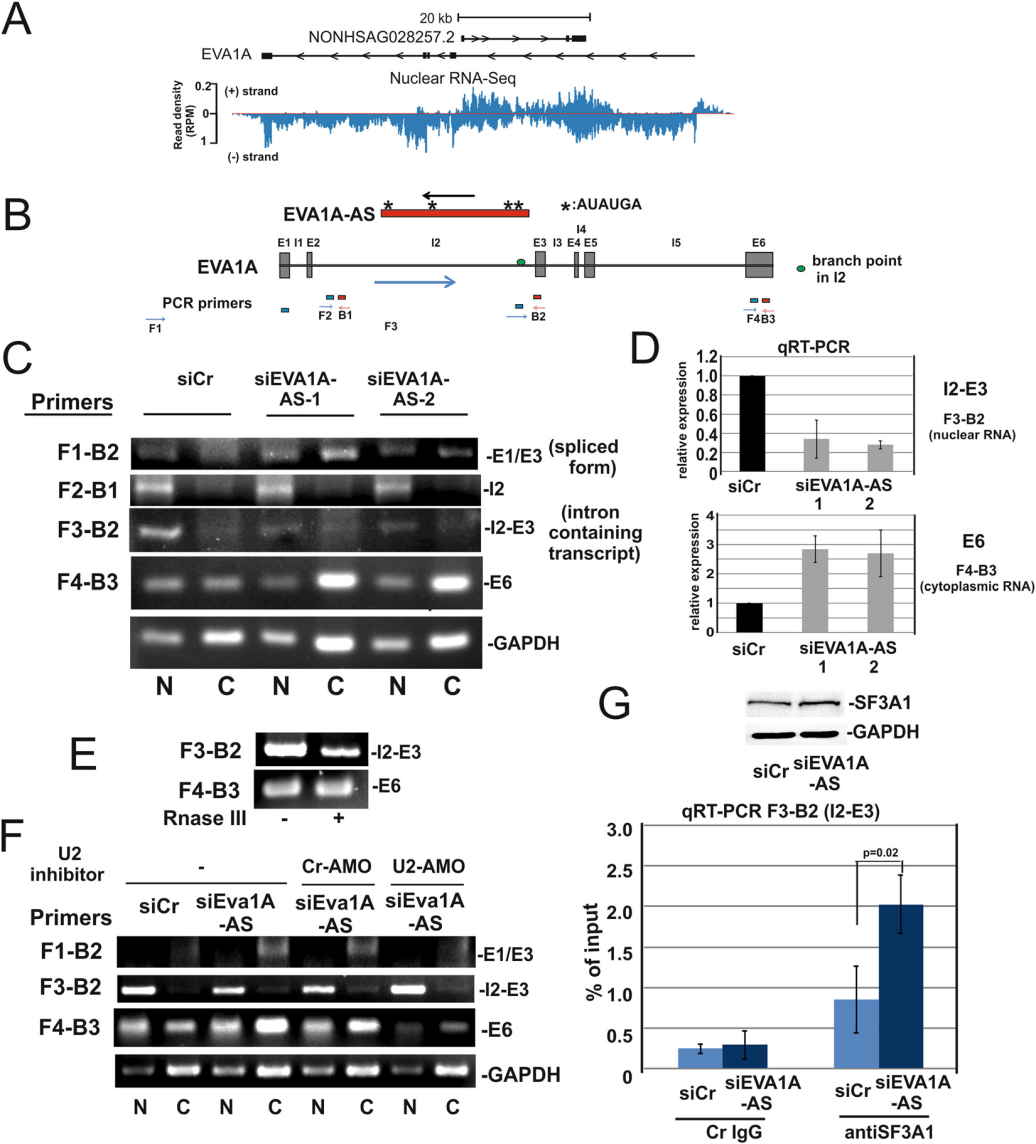
EVA1A-AS suppressed EVA1A expression by inhibiting the splicing of intron 2 of EVA1A. (**A**) Nuclear RNA-Seq data (ENCFF760IDU) from HepG2 generated by the ENCODE Consortium were aligned to the reference human genome (GRCh38). SeqMonk was used to quantitate and visualize the data. (+) strand: EVA1A-AS; (−) strand: EVA1A. (**B**) Scheme of EVA1A and EVA1A-AS. Positions of PCR primers are indicated (blue forward; red backward). (**C**) HepG2 cells were transfected with siCr, siEVA1A-AS-1 or siEVA1A-AS-2. RNAs were isolated from nuclear [N] or cytoplasmic [C] fractions and supplied for RT-PCR or qRT-PCR (**D**) as indicated. (**E**) Nuclear RNA was isolated from HepG2 cells, incubated with shortcut RNase III, and RT-PCR was performed using F3-B2 and F4-B3 primers. (**F**) Sister cultures from (**C**) were treated with Cr-AMO or U2-AMO and RT-PCR was then performed as described in (**C**). (**G**) HepG2 cells were transfected with siCr and siEVA1A-AS. Cell extracts were supplied for SF3A1 specific immunoblot or were incubated with control IgG or anti SF3A1 antibody and then precipitated with Protein G Sepharose. Bound RNAs were analyzed by EVA1A, I2-E3 specific qRT-PCR.

EVA1A-AS is transcribed in the opposite direction near the 3′ splice site of EVA1A I2 (Fig. 6A), suggesting that it may suppress the splicing of EVA1A I2. To examine whether EVA1A-AS exerts an influence on the EVA1A splicing event, we applied primer pairs located in Exon (E) 1, I2, E3 and E6 for RT-PCR or qRT-PCR in the siCr, siEVA1A-AS-1 or siEVA1-AS-2 treated HepG2 cells (Fig. 6B,C). When a primer pair that was located in E1-E3 (primers F1-B2) was used, we observed predominantly a 210 nt long PCR product instead of 298 nt in all samples. The 210 nt bands contain only E1 and E3 (Fig. 6C), implying that E2 is spliced out independently from EVA1A-AS in HepG2 cells. An intron containing EVA1A (using a primer pair located in I2 and E3) accumulated >3-fold in the nuclear fraction in siCr cells than in EVA1A-AS depleted cells (Fig. 6C). EVA1A mRNA is exported >2-fold in EVA1A-AS depleted cells than in siCr cells (Fig. 6D). These data suggest that EVA1A-AS impaired splicing of I2. We next analyzed the EVA1A-AS sequence to determine whether it could potentially form an RNA duplex with the U2 binding site. Notably, EVA1A-AS nascent RNA contains 4x UCAUAU (Fig. 6B) which are complementary to the branch point site sequence (AUAUGAU), predicted by branching point prediction (BPP)^11^ recognized by U2 snRNA in the I2 of EVA1A (Fig. 6B). To determine whether EVA1A and EVA1A-AS form a double-stranded RNA, we treated RNA from HepG2 cell with shortcut RNase III and then analyzed the undigested RNA. Upon treatment with RNaseIII, the amount of I2-E3 (intron containing transcript) was approximately 20% of that from the untreated RNA, while the amount of the E6 product was not changed (Fig. 6E), suggesting that I2 but not E6 RNA forms a double stranded RNA. Furthermore, treatment with the U2 inhibitor, U2-AMO (Gene Tools LLC, Oregon, USA), reduced the effect on I2 splicing by depletion of EVA1A-AS (Fig. 6F).

To further examine whether EVA1A-AS suppresses the recruitment of the U2 complex to the 3′splicing site of I2, we performed an RNA immunoprecipitation (RIP) assay using an antibody against the splicing factor 3a subunit 1 (SF3A1), a component of the mature U2 small nuclear ribonucleoprotein particle (snRNP), in siCr or siEVA1A-AS transfected cells. SF3A1 is expressed in similar amounts in both transfected cells (Fig. 6G), however using a primer pair located in I2 and E3 we showed that SF3A1 binds significantly stronger (p = 0.02) to EVA1A pre-mRNA in the absence of EVA1A-AS (Fig. 6G), suggesting that EVA1A-AS alters splicing of EVA1A by reducing the recruitment of U2 snRNP to EVA1A pre-mRNA.

## Discussion

The Myc gene has been implicated in the pathogenesis of most types of human cancerous tumors^12^. Notably, Myc induced not only pro oncogenic genes but also anti oncogenic genes. We have recently shown that Myc/Max dependent tumor suppressor miRNAs, miR9–5p and miR185-5p were inactivated via long noncoding RNA:Linc00176-sponge^6^. Linc00176 is also a Myc dependent gene^6^. In addition, some Myc inducible long noncoding RNAs participate in suppression of anti-oncogenic gene expression. We further examined additional Myc dependent antisense lncRNAs and their potential target coding RNAs that are downregulated in HCCs. Notably, in HepG2 cells at least 5 Myc dependent antisense noncoding RNAs are detected within Myc dependent coding genes, such as Flavin-containing monooxygenase (FMO)5^13^, zinc finger protein (ZFP) 69^14^, erythroblast membrane-associated protein (ERMAP) 26, and inhibitor of DNA binding (ID)2^15^. In addition, expression of these coding genes is suppressed in HCC, suggesting that these genes may negatively participate in cancer formation.

How does antisense RNA suppress splicing of sense RNA? It has been recently shown that several natural antisense transcripts (NATs) such as SAF and Zeb2-NAT lncRNAs altered the recruitment of members of the spliceosome to sense transcript by forming RNA-RNA duplexes with the sense transcript^16,17^. Here we showed that EVA1A-AS altered the recruitment of U2 snRNP to EVA1A pre-mRNA. Notably, EVA1A-AS nascent RNA contains 4x UCAUAU which are complementary to the branch point site sequence (AUAUGAU)^11^ recognized by U2 snRNP in the intron 2 of EVA1A. This implies that EVA1A-AS forms double-stranded RNA with EVA1A at the branch point site sequence and thus impair the recruitment of U2 snRNP.

EVA1A is a lysosome and endoplasmic reticulum-associated protein. Overexpression of EVA1A in non-small cell lung cancer cells induces cell cycle arrest at the G2/M phase^7^. EVA1A has also been shown to induce autophagosome formation^18^, and in glioblastoma, EVA1A inhibits cell proliferation by inducing autophagy and apoptosis^19^. Here it has been suggested that miR-125b plays a role in the resistance of HCC cells to chemotherapy via a mechanism involving the downregulation of EVA1A-mediated autophagy^20^. Autophagosome marker, LC3^21^, and EVA1A are only partially co-localized in HepG2 cells (SI Fig. 1B), and the molecular role of EVA1A in autophagosome formation and anti-proliferation in HCC is presently not clear. We show here, that overexpression of EVA1A causes microtubule catastrophe in G2/M phase. The microtubules play a role in autophagy formation, suggesting that microtubules formation may be influenced by EVA1A.

Notably, the depletion of EVA1A-AS caused the upregulation of endogenous EVA1A and concomitant accumulation of lipid droplets in the cytoplasm. Endogenous EVA1A localized with a similar distribution to that of lipid droplets. It has been shown that in the budding yeast system lipid droplets maintain lipid homeostasis during anaphase for efficient cell separation^22^. Upon treatment with CDK1 inhibitor RO3306, lipid droplets accumulated in the cytoplasm within 24 h, suggesting that lipid droplet accumulation may be an indirect effect of EVA1A. The exact relationship between EVA1A, lipid droplets, and HCC mitosis still remains to be studied.

Although 161 putative driver genes are associated with 11 recurrently altered pathways in HCC development^23^, these mutations were not observed in chronic hepatitis or cirrhosis (preneoplastic stages). Interestingly, 28% of the altered gene products play a role in chromatin remodeling, suggesting that a subset of genes expresses only in HCC but not in normal hepatocytes^23^. Conversely, genes that are expressed in normal liver were not expressed in HCC. Here, products from coding RNAs that are targets of long antisense noncoding RNA may be suitable negative biomarkers for early stages of HCC. Since many long noncoding RNAs are expressed in cell type specific manner, long antisense noncoding RNAs in cancer cells may be promising targets for therapy as well as useful tools to detect novel cancer type specific negative biomarkers.

## Methods

### Cell culture, siRNA, and transfection

HepG2 and Huh7 cells were grown in DMEM supplemented with 10% FCS. RK-13 cells were grown in EMEM supplemented with 10% FCS.

siMyc (sc-29226) was from Santa Cruz technology (Santa Cruz, CA, USA). Control siRNA-1 (5′-UAAGGCUAUGAAGAGAUAC-3′), siMax (5′-GAAUUGUCUUGCAAGUUAA-3′), siMxi1 (5′-CACUUGGUUUGCUCAACAA-3′), siEVA1A (5′-CCUUUUUGGCCAAAU GUAU-3′), siEVA1A-AS-1 (5′-UCCUCUAAUUCAGAAAUUUCU-3′), and siEva1-AS-2 (5′-CUCCAGUAUAACAUAAAGUGU-3′) were from Microsynth AG (Balgach, Switzerland). GFP-EVA1A and Myc tagged EVA1A plasmids were kindly provided by Prof. Chen (Peking University, China)^18^. Plasmid pmRFP-LC3^24^ was from Addgene (Cambridge, MA, USA). Lentivirally modified shTHOC5 and shCr (non-targeting control) HepG2 cells were described previously^5^.

### RNA sequencing and data analysis

RNA-seq libraries were generated using the NEB Next Ultra Directional RNA Library Prep Kit for Illumina (NEB). Poly-A-tailed mRNA was isolated from total RNA using oligo-dT beads. Purified mRNA was then fragmented with heat in fragmentation buffer. First strand and second strand cDNA syntheses were performed in accordance with the manufacturer’s recommendations. Second strand cDNA was then end-repaired, ligated to an NEBNext Adaptor and individually indexed, followed by limited-cycle (10) amplification. Indexed libraries were pooled and sequenced on an Illumina HiSeq 2500. FASTQ files were generated by CASAVA (v1.8.2). Galaxy workflow for RNA-Seq (www.usegalaxy.org) was used for subsequent data analysis. Reads were mapped to the human reference genome (GRCh38) using Bowtie2 (Galaxy Version 2.3.4.1). The gene expression values (Fragments Per Kilobase Million (FPKM)) were calculated by Cuffdiff (Galaxy Version 2.2.1.5) using the human NONCODEv5 transcript reference. The complete RNA-seq data along with processing protocols have been deposited in NCBI’s Gene Expression Omnibus and is accessible through GEO series accession number GSE115139.

### Chromatin immunoprecipitation (ChIP)

ChIP experiments were performed as previously described^25^. Briefly, in a confluent 10-cm dish, HepG2 cells were crosslinked in 1% formaldehyde for 5 min at 37 °C in the presence of 5% CO_2_. Cross-links were quenched in 125 mM glycine^25^. Cells were washed 3x in ice-cold PBS. Nuclei were fractionated, resuspended in sonication buffer (15 mM Tris-HCl pH 8.0, 0.1% SDS and 2 mM EDTA) and then sheared into 500-bp DNA fragments using Covaris AFA (Adaptive Focused Acoustics, Woburn, MA, USA) technology according to the manufacturer’s instructions^25^. Aliquots of extracts were precipitated pre-coated Protein G Agarose-PLUS (Santa Cruz Biotechnology Inc.) with control IgG, 3 µg anti Myc (Santa Cruz Biotechnology Inc.), or Max (Proteintech group, IL, USA) antibodies. Following 2 h at 4 °C, the beads were washed three times in RIPA buffer, and two times in wash buffer (500-mM NaCl, 1% NP40, 100-mM Tris–HCl at pH 8)^26^. Cross-links were reversed for 6 h at 65 °C (250 mM-NaCl)^25^. One microliter of RNase A (100 mg/ml) was added to the bead suspension and incubated for 10 min at RT. Following proteinase K digestion at 55 °C for 1 h, the bound DNA fraction was isolated using NucleoSpin Extract II (Macherey-Nagel, Dueren, Germany)^25^. PCR was then performed using EVA1A or EVA1A-AS promoter-specific primers as shown in Table 1 (ChIP).

**Table 1:** PCR primer pair sequences for selected genes.

Gene	Accession number	Forward primer	Reverse primer	RT-PCR	qRT-PCR	ChIP	PCR product (bp)
*EVA1A*	NM_001135032.1	ATGCCCCTTTTCTAGCCAGG	CAGTCGCGTTTCTCCGATCT	x	x		153
*EVA1A-AS*	NR_110281.1	CCTGCATCACTGCATTTCCG	TGCGAAAGAGTGGCACACAG	x	x		145
*GAPDH*	NM_002046	TGTTGCCATCAATGACCCCTT	CTCCACGACGTACTCAGCG	x	x		202
*ID1*	NM_002165.3	CTCTGCACACCTACTAGTCACC	GTCACGTTTGGTGCTTCTCAGATTTC		x		117
*Max*	NM_002382	CAATCTGCGGCTGACAAACG	GCACTTGACCTCGCCTTCT	x			272
*Mxi1*	NM_130439	GCGCCTTTGTTTAGAACGCTT	AATGCTGTCCATTCGTATTCGT	x			235
*Myc*	NM_002467.4	CATGAGGAGACACCGCCCACCACCAG	TGCGTAGTTGTGCTGATGTGTGGAGACGTG	x			210
*SPC25*	NM_020675	TCATCTTGAGGGCCTAGCAG	CATTGTGCACATGTACCCTAAAAC		x		221
*THOC5*	NM_001002878	GAGACCCTCACCAGCAAACAC	CGAATGGCATAAACAGGTACTCC		x		213
*F1-B2*		TTTGTCGTGTCCGCCCCTCAG	TGTCAGGAGCAGAGAAGTTTCTG	x			210
*F2-B1*		CTGGACATAGTGGCATATAACTGTAG	GATGATGAACTCAGTTTCAGATATGTTG	x			242
*F3-B2*		GTATGACCCTGGGAGACTGG	TGTCAGGAGCAGAGAAGTTTCTG	x	x		329
*F4-B3*		ATGCCCCTTTTCTAGCCAGG	CAGTCGCGTTTCTCCGATCT	x	x		153
*EVA1A promoter*		GCCGTTCAGGTTTCGCGTCTT	CAAGCCCCGCAGAAGCTGCA			x	176
*EVA1A-AS promoter*		CTCACTCTCCAATCGGAACCTCTA	CTCATGAGAACTCACCCACTATTAG			x	193

EVA1A: eva-1 homolog A, regulator of programmed cell death; EVA1A-AS: eva-1 antisense RNA; GAPDH: glyceraldehyde-3 phosphate dehydrogenase; ID1: inhibitor of DNA binding 1, HLH protein; Max: MYC associated factor X; Mxi1: MAX-interacting protein 1; Myc: v-myc avian myelocytomatosis viral oncogene homolog; SPC25: NDC80 kinetochore complex component; THOC5: THO complex subunit 5 homolog.

### Reporter assay

Promoter regions of EVA1 (−1–478) and EVA1A-AS (−1–988) were cloned into pGL2-basic vector. pcDNA3.1+/C-(K)-DYK-MYC was purchased from Genscript (NJ,USA). For luciferase assays, RK-13 cells were seeded on a 96-well plate and 0.5 μg of plasmids were cotransfected using Lipofectamine 2000 (Thermo Fisher Scientific, MA, USA). The cells were incubated for 36 h in complete medium. Luciferase assay was performed using Steady-Glo Luciferase Assay System (Promega) according to manufacturer’s protocol. β-galactosidase activities were measured using Beta-Glo Assay System (Promega). The relative luciferase activities were normalized to the β-galactosidase activities.

### Propidium isodide labelling followed by FACS analysis

HepG2 cells were trypsinized and fixed in ice-cold ethanol. Then fixed cells were then stained in propidium iodide (PI) solution (100 µg/ml PI, 100 µg/ml RNAse, 0.05% Triton X-100) and analysed by Accuri-C6 flow cytometer (BD Biosciences) under standard settings for detecting PI (FL-2) using a 488 nm laser.

### Immunofluorescence and immunohistochemistry

Immunofluorescence was performed as previously described^27^. TUNEL staining using *in situ* cell death detection kit (Roche Diagnostics, Mannheim, Germany) was performed according to the manufacturer’s instructions. Counterstaining was performed using 4′,6-diamidin-2-phenylindole (DAPI). Immunohistochemical studies were performed as detailed previously^5^. Rabbit monoclonal anti Ki67 was purchased from Thermo Sientific (MA, USA).

### Immunoblotting procedures

Details of immunoblotting have been described previously^28^. Monoclonal antibody against GAPDH was purchased from Santa Cruz Biotechnology (Santa Cruz, USA). Rabbit polyclonal anti EVA1A antibody was obtained from MyBioSource.Inc (San Diego, CA, USA).

Corresponding proteins were visualized by incubation with peroxidase conjugated anti-rabbit or anti-mouse immunoglobulin (Santa Cruz Biotechnology) followed by incubation with SuperSignal West FemtoMaximum Sensitivity Substrate (Pierce, Rockford, IL, USA). Results were documented on a LAS4000 imaging system (GE Healthcare Bio-Sciences, Uppsala, Sweden)^6,29,30^.

### Semi-quantitative RT-PCR and qRT-PCR analysis

Human normal hepatocyte RNA was purchased from Origene (Maryland, USA). RNA was isolated from cells with the High Pure RNA Isolation kit (Roche Diagnostics) according to the manufacturer’s instructions^25,27,28^. 1 µg of RNA was reverse-transcribed using oligo dT primer and the Omniscript reverse transcriptase kit (Qiagen, Hilden, Germany) following the instructions provided. One-twentieth of the cDNA mix was used for real-time PCR using 10 pmol of forward and reverse primer and ORA qPCR Green Rox kit (HighQu, Kraichtal, Germany) in a Qiagen Rotorgene machine^25^. The levels of mRNA expression were standardized to the glyceraldehyde-3 phosphate dehydrogenase (GAPDH) mRNA level^25^. Primer pairs for each PCR are described in supplemental information Table 1.

### RNA immunoprecipitation (RIP) assay

SF3A1– EVA1A-AS complex: HepG2 cells were lysed with lysis buffer (10 mM Tris, 150 mM NaCl, 1 mM PMSF, 0.5% NP40, protease inhibitor cocktail (Sigma-Aldrich) and RNase inhibitor)^31^. After centrifugation, supernatants were incubated with control IgG or anti SF3A1 antibody (Bethyl, TX, USA), and then precipitated with Protein G Sepharose. Bound RNAs were analyzed by RT-PCR^27^.

### Double-stranded RNA assay

The nuclear fraction from HepG2 cells was suspended in 200 µl of RIPA buffer (150 mM NaCl, 1% NP40, 0.1% SDS, 20 mM MnCl_2_, 50 mM Tris-Cl at pH 8, 5 mM EDTA at pH 8) and then frozen and thawed three times. After centrifugation, 10 units of Shortcut RNAse III (NEB) which specifically digests double-stranded RNA were added and incubated at 37 °C for 30 minutes. RNAs were then isolated using the ReliaPrep miRNA Cell and Tissue Miniprep System according to the manufacturer’s protocol. cDNA synthesis was carried out using ProtoScript II First Strand cDNA Synthesis Kit (NEB) and Oligo d(T)/ random primers mix (NEB).

### TCGA data

Liver Hepatocellular Carcinoma (TCGA, Provisional cohort) was used for the study. Gene expression quantification data of primary HCCs were downloaded from “GDC Data Portal” (https://portal.gdc.cancer.gov/). Mutation was analyzed using an online tool of the GDC portal.

### Statistical analysis and limitation of the study

Cell experiments were performed in triplicate and a minimum of three independent experiments were evaluated^6,25,32^. Data were reported as the mean value +/− standard deviation (SD)^6,27^. The statistical significance of the difference between groups was determined by the Student’s test (two-sided)^6^. Primary 366 HCC data gathering was limited by the availability from the cancer genome atlas (TCGA) data (https://cancergenome.nih.gov/)^6^.

## Supplementary information

Supplementary information

## Data Availability

All data generated or analyzed during this study are included in this published article and its additional files.
