# Supplementary material for: Myc/Max dependent intronic long antisense noncoding RNA, EVA1A-AS, suppresses the expression of Myc/Max dependent anti-proliferating gene EVA1A in a U2 dependent manner
Source: Sci Rep. 2019 Nov 21;9:17319. doi: 10.1038/s41598-019-53944-2 (PMC6872820; doi:10.1038/s41598-019-53944-2)

Supplementary Information

Myc/Max dependent intronic long antisense noncoding RNA, EVA1A-AS, suppresses the expression of Myc/Max dependent anti-proliferating gene EVA1A in a U2 dependent manner

Svenja E Niehus [1#], Aldrige B Allister[1#], Andrea Hoffmann[2], Lutz Wiehlmann [3], Teruko Tamura [1], and Doan Duy Hai Tran[1*]

1 Institut fuer Biochemie, OE4310, Medizinische Hochschule Hannover, Carl-Neuberg-Str. 1, D-30623 Hannover; Germany, 2 Klinik für Orthopädie OE8893, Medizinische Hochschule Hannover, Stadtfelddamm 34, D-30625 Hannover, Germany. 3. Zentrale Forschungseinrichtung Genomics OE 9415, Medizinische Hochschule Hannover, Carl-Neuberg-Str. 1, D-30623 Hannover; Germany

* Correspondence to: Dr. Doan DH Tran : Institut fuer Zellbiochemie, OE4310, Medizinische Hochschule Hannover, Carl-Neuberg-Str. 1, D-30623 Hannover, Germany Tel: +49-511-532-2857 , E-mail: Tran.Doan@MH-Hannover.de

# These authors contributed equally to this work.

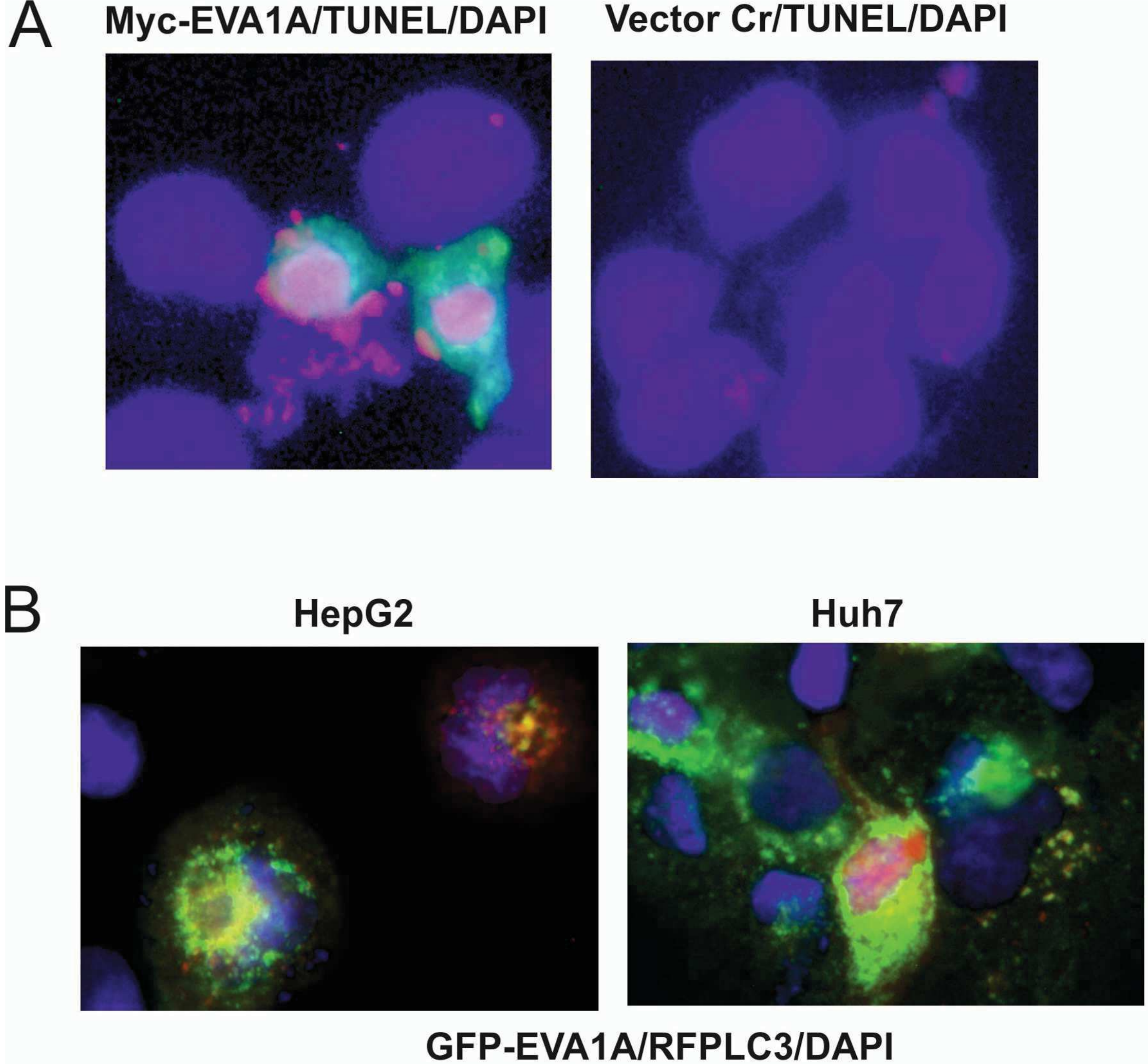

Niehus et al. SI Figure 1

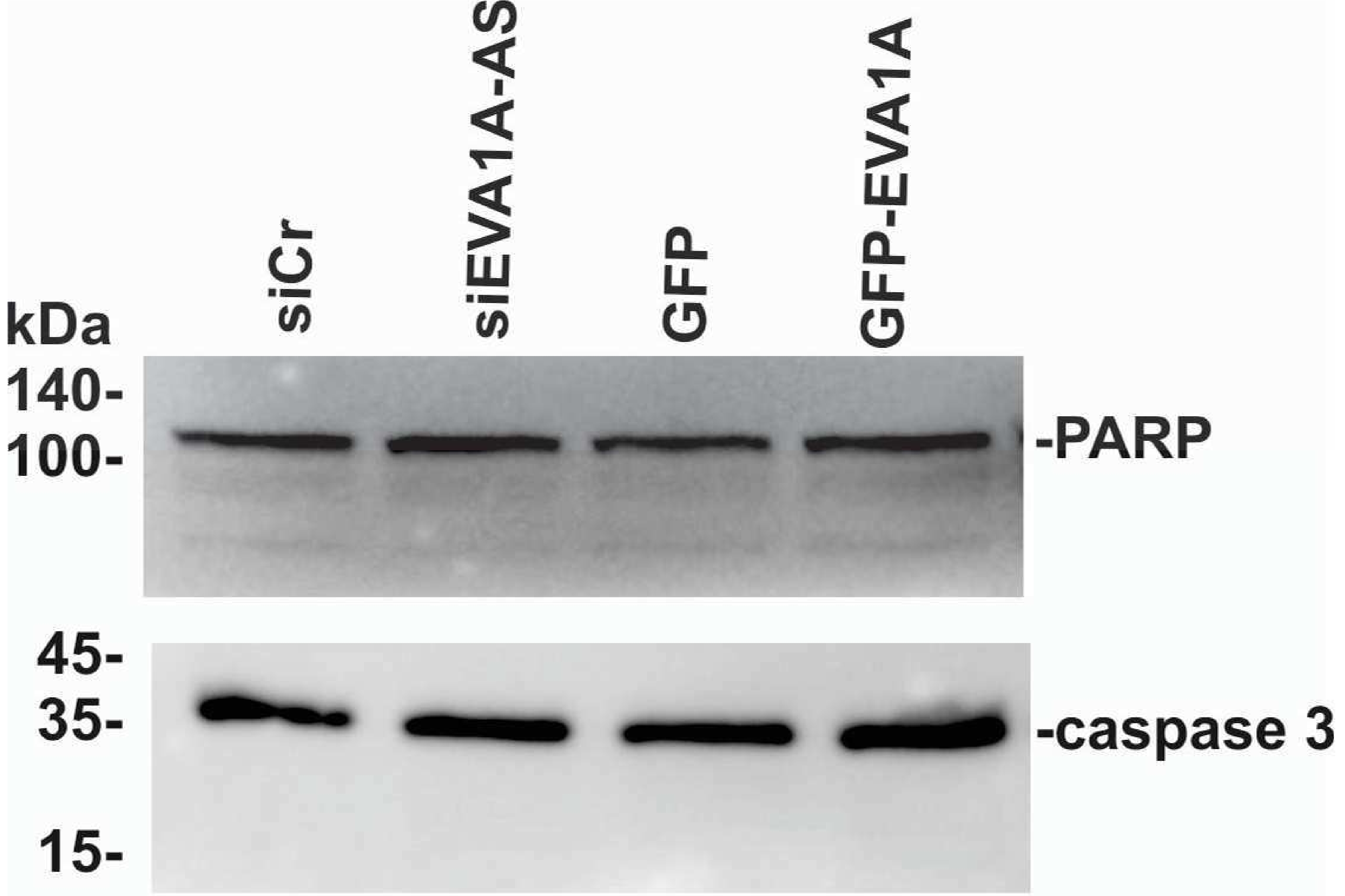

Niehus et al. SI Figure 2.

Raw data

Supple. Fig. 1A

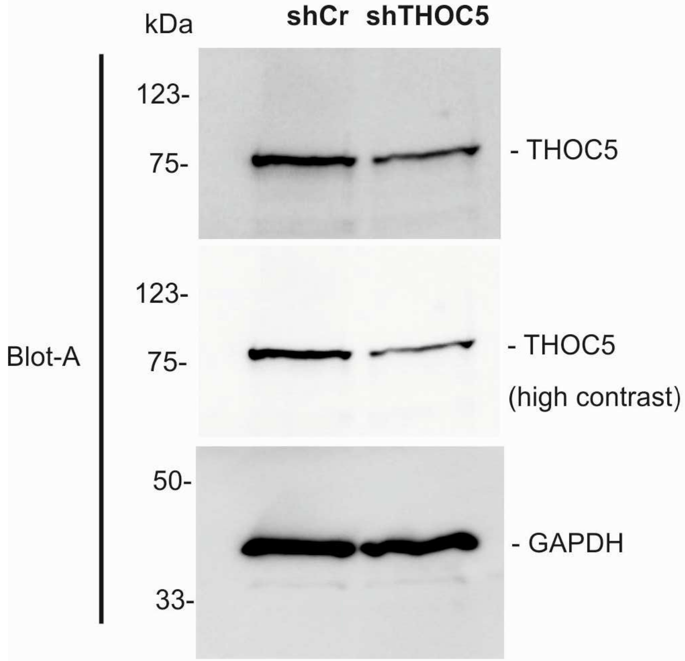

Supple. Fig. 1D

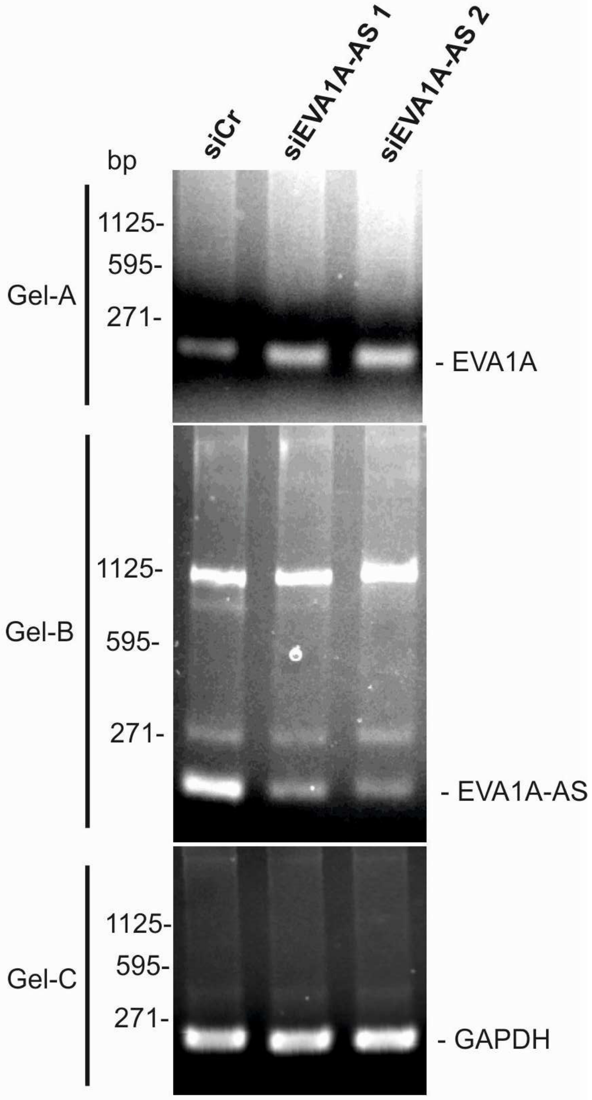

Supple. Fig. 1E

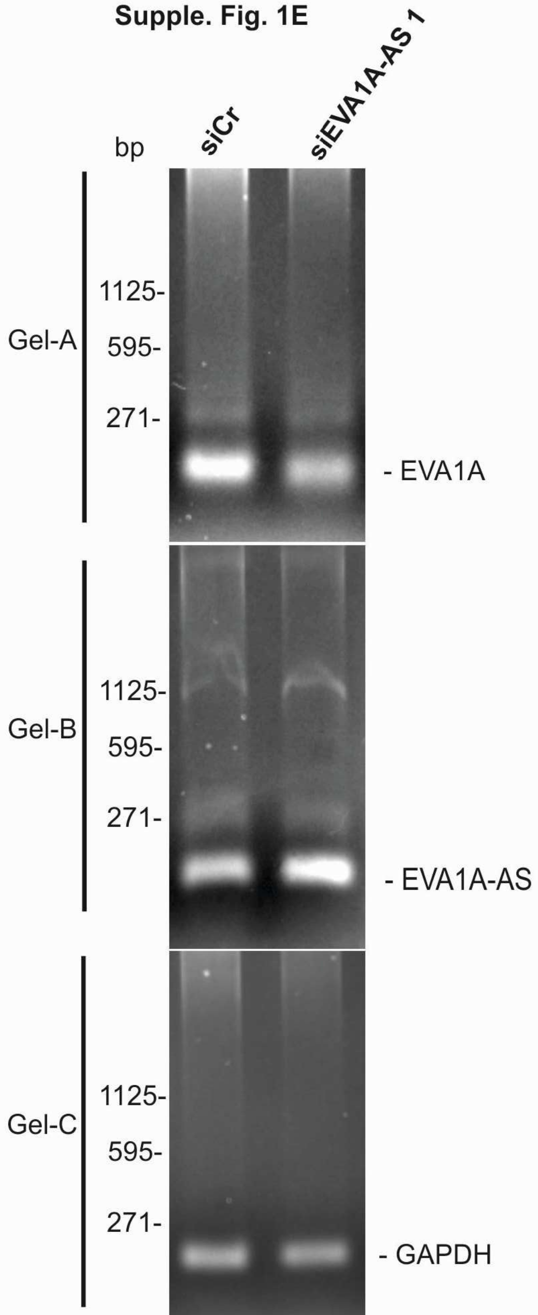

Supple. Fig. 1I

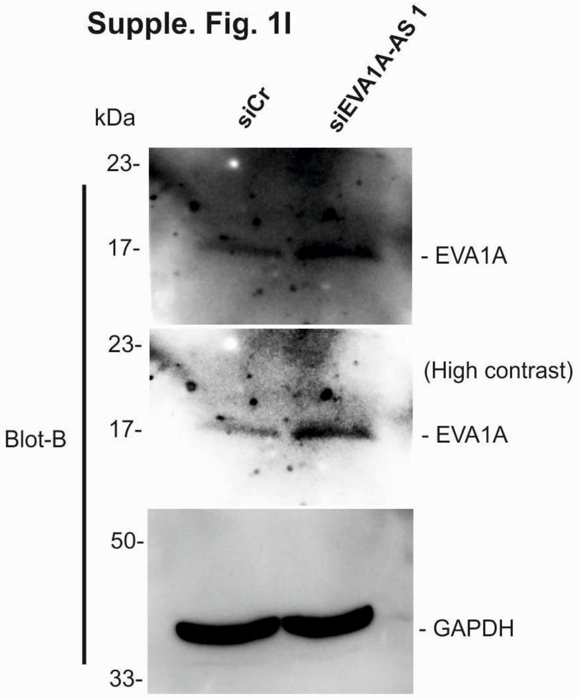

Supple. Fig. 3C

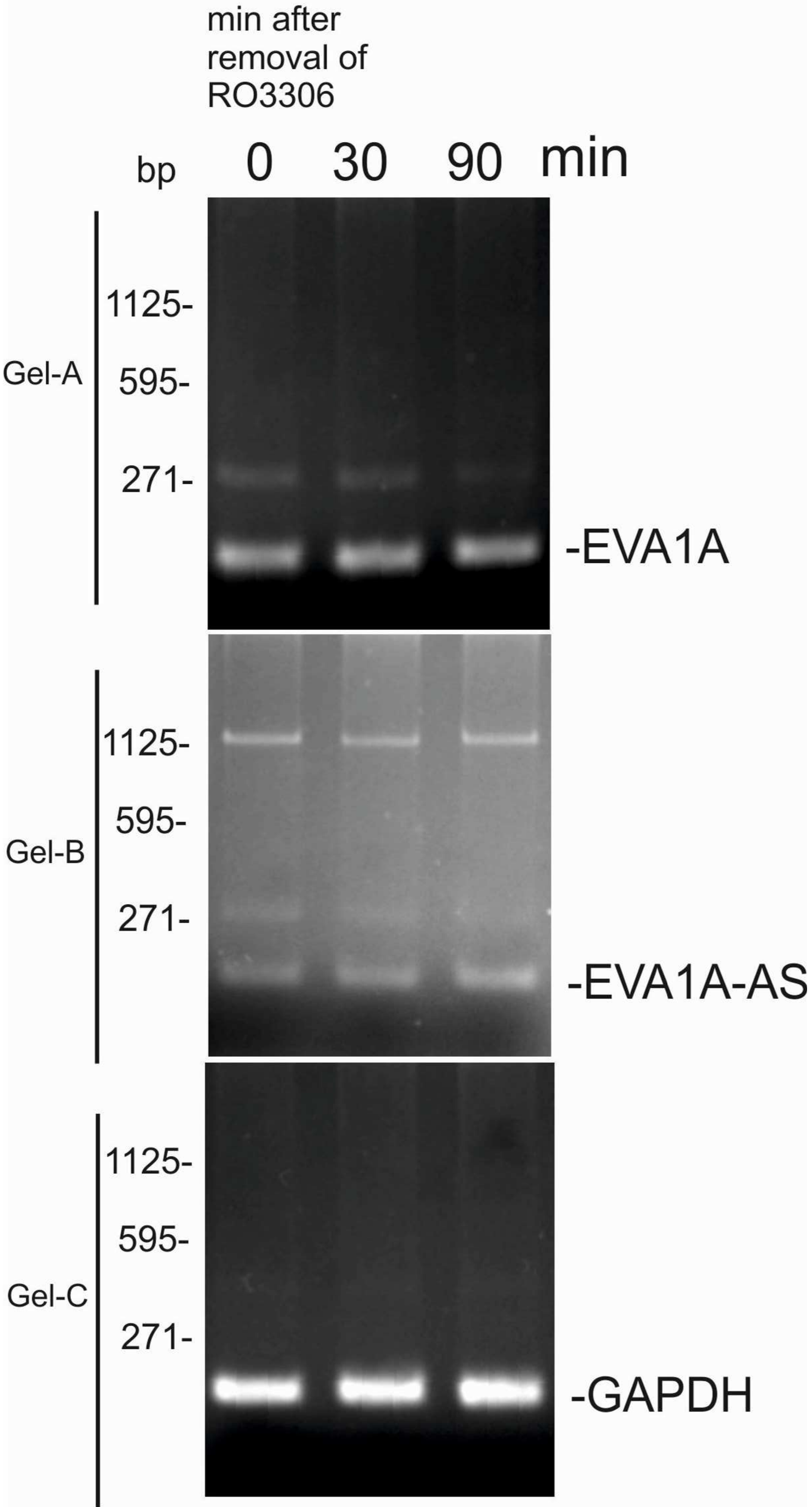

Supple. Fig. 4A

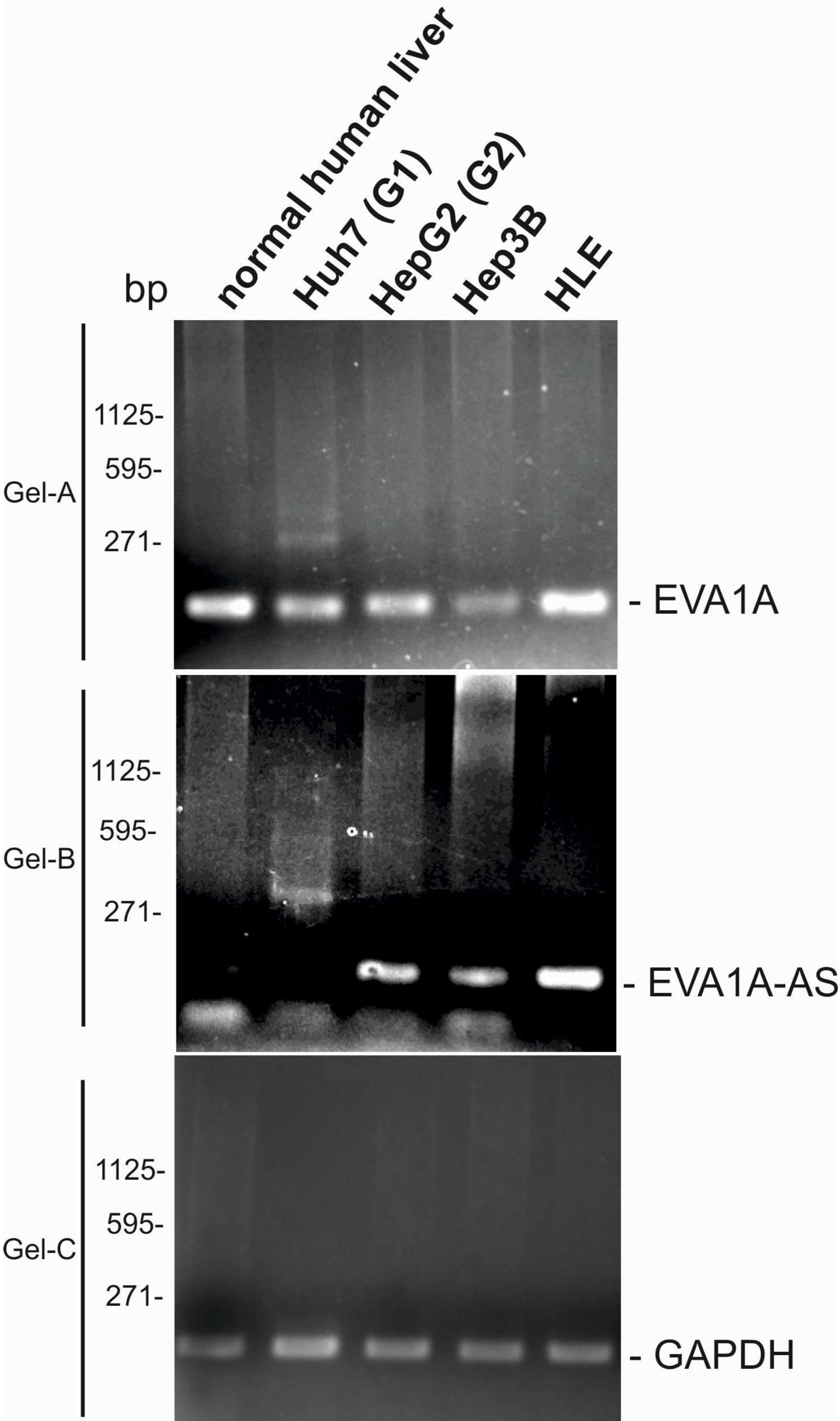

Supple. Fig. 5B

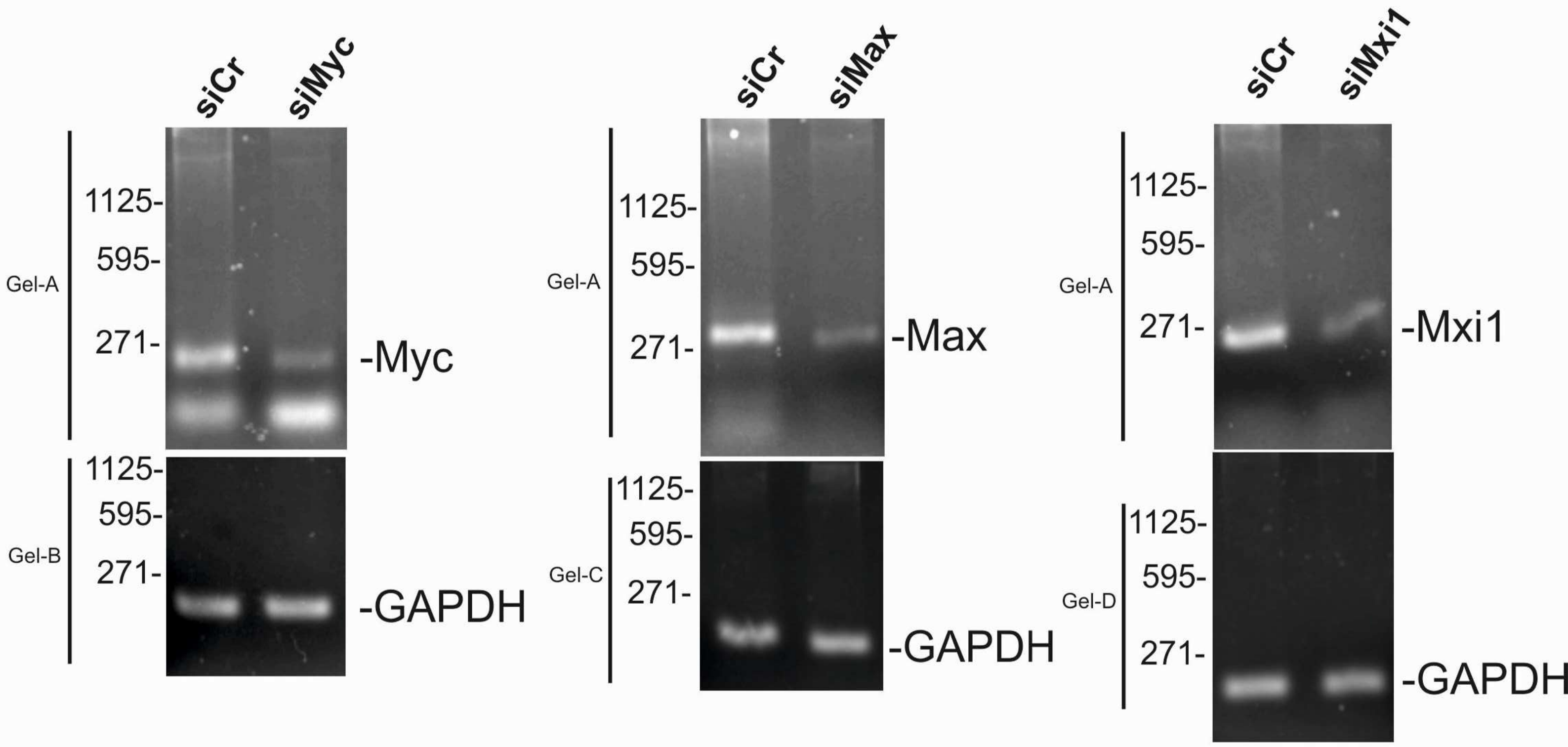

Supple. Fig. 5C

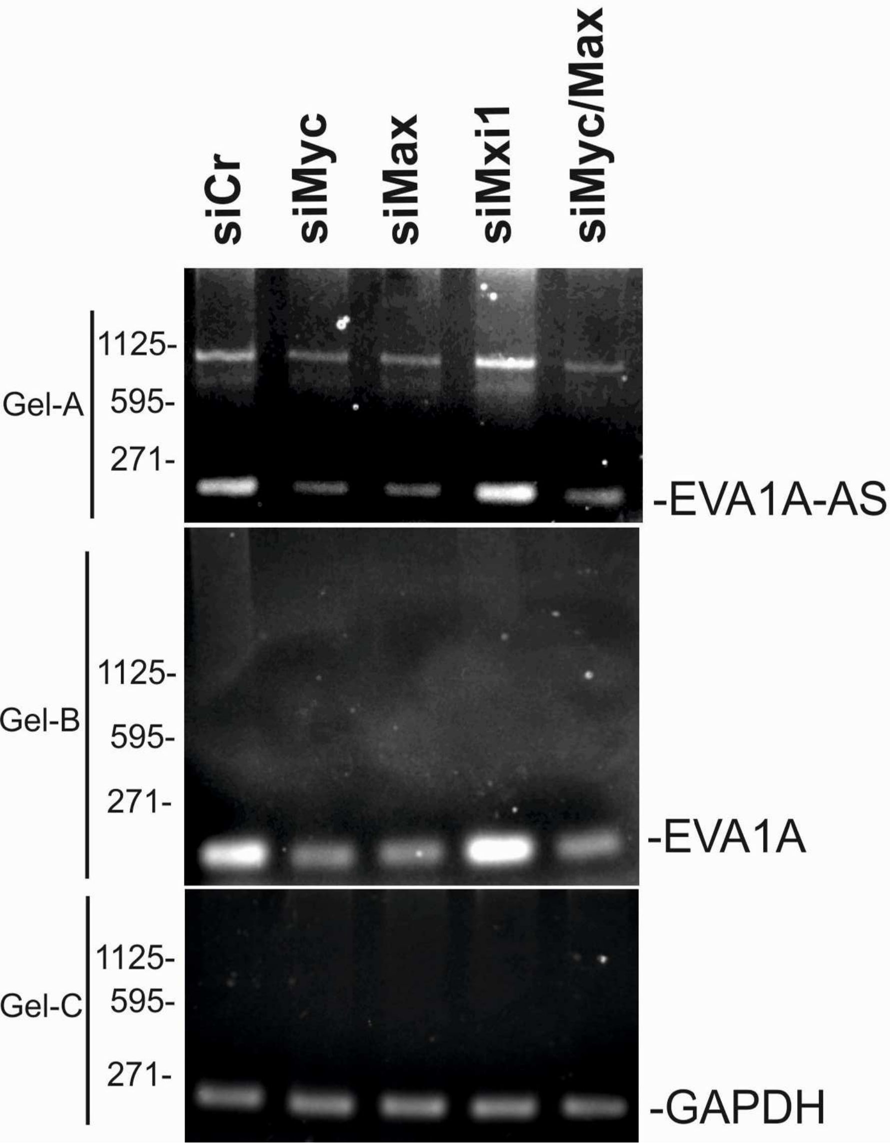

Supple. Fig. 6C

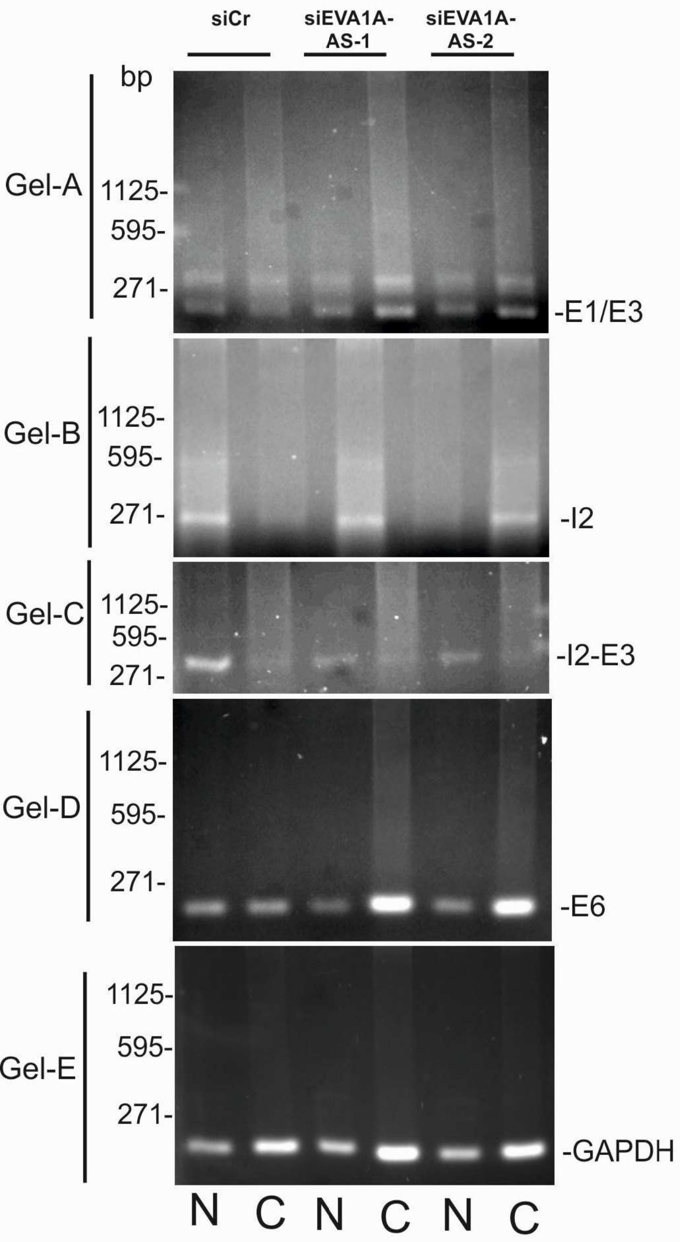

Supple. Fig. 6E

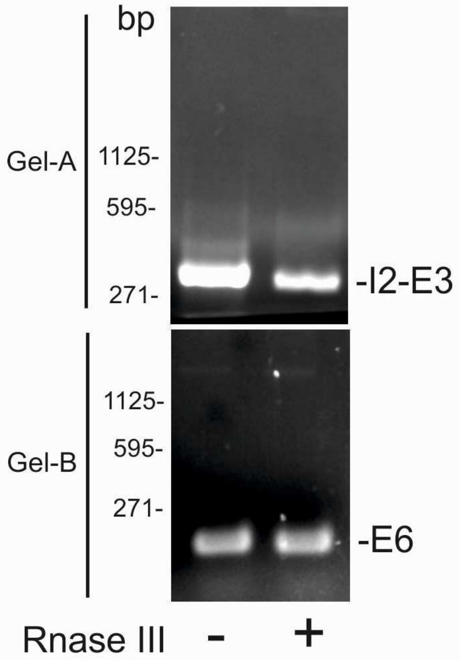

Supple. Fig. 6G

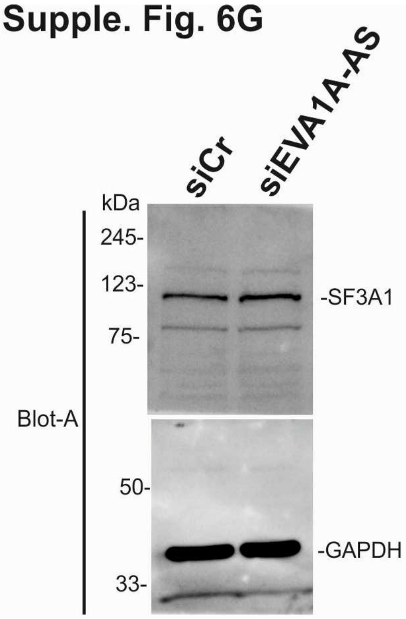

Supple. Fig. 6F

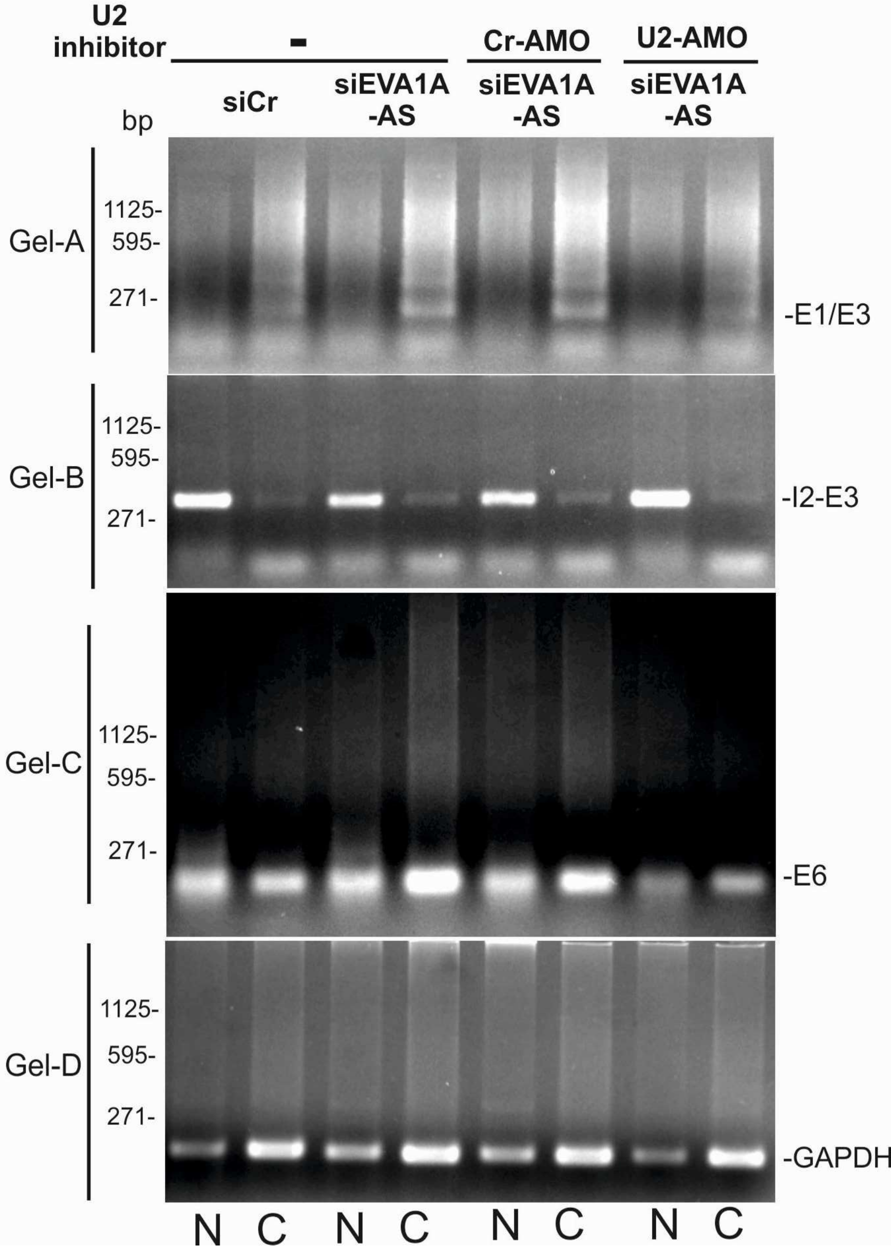

Supplement: Supplementary file 1 — Supplementary information [file 41598_2019_53944_MOESM1_ESM.pdf]
